# The airways microbiome of individuals with asthma treated with high and low doses of inhaled corticosteroids

**DOI:** 10.1371/journal.pone.0244681

**Published:** 2020-12-30

**Authors:** Matthew J. Martin, Nur Masirah M. Zain, Glenn Hearson, Damian W. Rivett, Garrit Koller, David J. Wooldridge, Graham Rose, Saheer E. Gharbia, Ben Forbes, Kenneth D. Bruce, Tim W. Harrison

**Affiliations:** 1 The Asthma Centre, Nottingham Respiratory Research Unit, University of Nottingham, Nottingham City Hospital, Nottingham, United Kingdom; 2 Faculty of Life Sciences & Medicine, School of Cancer & Pharmaceutical Sciences, Institute of Pharmaceutical Science, King’s College London, London, United Kingdom; 3 National Infection Service, Public Health England, London, United Kingdom; University of Rome, ITALY

## Abstract

**Background:**

Inhaled corticosteroids (ICS) are the mainstay of asthma treatment, but evidence suggests a link between ICS usage and increased rates of respiratory infections. We assessed the composition of the asthmatic airways microbiome in asthma patients taking low and high dose ICS and the stability of the microbiome over a 2 week period.

**Methods:**

We prospectively recruited 55 individuals with asthma. Of these, 22 were on low-dose ICS and 33 on high-dose ICS (16 on budesonide, 17 on fluticasone propionate). Sputum from each subject underwent DNA extraction, amplification and 16S rRNA gene sequencing of the bacterial component of the microbiome. 19 subjects returned for further sputum induction after 24 h and 2 weeks.

**Results:**

A total of 5,615,037 sequencing reads revealed 167 bacterial taxa in the asthmatic airway samples, with the most abundant being *Streptococcus* spp. No significant differences in sputum bacterial load or overall community composition were seen between the low- and high-dose ICS groups. However, *Streptococcus* spp. showed significantly higher relative abundance in subjects taking low-dose ICS (p = 0.002). *Haemophilus parainfluenzae* was significantly more abundant in subjects on high-dose fluticasone propionate than those on high-dose budesonide (p = 0.047). There were no statistically significant changes in microbiota composition over a 2-week period.

**Discussion:**

Whilst no significant differences were observed between the low- and high-dose ICS groups, increased abundance of the potential pathogen *H*. *parainfluenzae* was observed in patients taking high-dose fluticasone propionate compared to those taking high-dose budesonide. The microbiota were stable over fourteen days, providing novel evidence of the established community of bacteria in the asthmatic airways.

**Clinical trial registration:**

ClinicalTrials.gov NCT02671773

## Introduction

Asthma is a common cause of morbidity and mortality affecting an estimated 334 million individuals worldwide [1, 2]. In the UK, a stepwise approach to patient management recognises five different stages reflecting severity and corresponding treatment strategies. All stages now include inhaled corticosteroids (ICS) [3–5] which are also commonly prescribed in chronic obstructive pulmonary disease (COPD) [6]. Although generally regarded as safe, considerable evidence suggests their use is associated with an increased risk of respiratory infection in COPD [6], and there are suggestions this may also be the case in asthma [4, 5] although not all studies are in agreement [7].

The role of bacteria in the pathophysiology of asthma is not fully established or well-defined [8, 9]. Fundamentally however, the key initial step towards a better understanding is characterising the abundance and diversity of bacteria in the asthmatic airways. This is now possible due to techniques that characterise microbiome composition. Previous studies have described bacterial species in the lower respiratory tract of individuals with asthma as well as making tentative associations with disease severity [10, 11]. In compositional terms, the most common genera across many microbiome studies of the asthmatic airways are *Prevotella*, *Veillonella*, *Haemophilus*, *Streptococcus* and *Moraxella* [12–15]. Some of these genera contain potential pathogens which are common causes of pneumonia, such as *Haemophilus* and *Streptococcus* spp. and both of these species have been detected at higher frequencies in poorly controlled asthmatics and subjects with treatment-resistant severe asthma [12, 14–16].

Here, we report the culture-independent analysis of bacteria present in sputum samples of 55 adults with asthma. Data were collected on the composition of the bacteria forming the microbiome and the total bacterial and taxa-specific loads in the samples were quantified. Our aims were to assess whether long-term use of different doses and types of ICS were associated with differences in microbiome composition in asthma and the short-term stability of the microbiome.

## Methods

### Participants

Fifty-five adults (age ≥18) were recruited from Nottingham City Hospital outpatient respiratory clinics or from an existing research subject database. All subjects provided written, informed consent to the study which was approved by the National Research Ethics Committee East Midlands–Derby 1 (Ref 14/EM/0091).

The study included adult patients who had not smoked for ≥10 years and had a <10 pack year smoking history. All participants had a previous physician diagnosis of asthma (see S1 File) with no other respiratory diagnosis and no respiratory infection or antibiotic administration in the month prior to the study. Subjects with a post-bronchodilator FEV_1_ of <60% were excluded for safety reasons due to the use of nebulised hypertonic saline. Recruitment targeted 20 patients on low-dose ICS (beclomethasone dipropionate (BDP) ≤400 μg/day, fluticasone propionate (FP) ≤200 μg/day or budesonide (BUD) ≤400 μg/day for at least a year) and 30 taking high-dose ICS (FP ≥500 μg/day or BUD ≥800 μg/day for at least a year) with approximately equal numbers taking FP and BUD with or without long-acting β_2_-agonist.

### Clinical measurements

At visit 1 demographic details and medical history were recorded. Spirometry was performed and the Asthma Control [17] and Leicester Cough Questionnaires [18] administered. Exhaled nitric oxide (FE_NO_) measurement was performed prior to sputum induction. A subgroup of 20 patients agreeable to further visits were asked to return for repeat sputum induction after 24 h and 2 weeks (see S1 File). This subgroup of patients were asked about changes in symptoms or signs of active infection prior to each sputum induction and FEV_1_ prior to sputum induction was assessed on each occasion.

### Sample collection

Induced sputum samples were obtained from patients using 3, 4 & 5% hypertonic saline as previously described [19, 20]. Samples were frozen at -80°C pending microbiological analysis.

### Culture-independent analyses

#### DNA extraction

Sputum samples were disrupted as previously described [21]. Processing followed recommendations for Gram-positive bacterial DNA extraction (GenElute™ Bacterial Genomic DNA Kit) (see S1 File).

#### 16S rRNA gene sequencing

16S rRNA gene sequencing (V3-V4 region) was performed on the Illumina MiSeq platform. Library preparation was carried out according to Illumina 16S Metagenomic sequencing library preparation manual (see S1 File). The paired end reads were rarefied to 9311 reads and transformed into Operational Taxonomic Units (OTU). This was determined using the QIIME version 1.9.1 pipeline to cluster the 16S rRNA gene sequences.

#### qPCR

Total bacterial load qPCR was applied as previously described [22]. Two species-specific qPCR assays were used to quantify the load of *Haemophilus influenzae* [23] and *Streptococcus pneumoniae* [24] cells (see S1 File).

### Statistical analyses

#### Microbiota analysis

To identify multiple interactive effects, normalised OTU relative abundance (%) were multiplied by their corresponding qPCR-derived total bacterial load obtained from the qPCR data to give a CFU/ml value. Diversity indices were calculated from bootstrapped OTU tables using the vegan package of R (https://cran.r-project.org/web/packages/vegan/vegan.pdf) such as richness, Shannon’s and Simpson’s. Prior to analyses, data were assessed for homogeneity of variance and the normal distribution of the residuals. Non-metric multidimensional scaling (NMDS) using Bray-Curtis distances assessed overall compositional dissimilarity. Analysis of Similarity (ANOSIM) was used to identify differences between the groups. The effect of treatment on individual OTU abundance was analysed using non-parametric Wilcoxon rank sum tests with Bonferroni corrections for multiple comparisons.

#### Quantitative bacterial load analysis

Quantitative PCR data (CFU/ml) were logarithm (base = 10) transformed prior to analysis (SPSS Statistics v23.0; IBM Inc., USA). Same-day data were analysed based on a non-pairwise comparison (independent T-test or Mann-Whitney test). Data from different timepoints were compared using repeated measures ANOVA.

## Results

### Subjects

Induced sputum samples were collected from 55 subjects with asthma (Table 1). Twenty-two and 33 subjects were treated with low- and high-dose ICS, respectively (Tables 1 and 2). In the low-dose group, 10 subjects were taking BDP, 7 BUD and 5 FP; whereas in the high-dose group, 16 subjects were taking BUD and 17 subjects FP (Table 2). None of the subjects were taking LAMAs and 9 in the low-dose group and 30 in the high-dose group took concomitant LABAs. Repeat sputum samples (after 24 h and 2 weeks) were collected from 19 subjects, 8 of whom were taking low-dose ICS and 11 high-dose ICS (Table 2). None of the 19 subjects reported any change in symptoms prior to repeat sputum induction at visits 2 and 3 and all FEV_1_ measurements at visits 2 and 3 were within 150ml of the baseline values.

**Table 1:** Study participant characteristics.

	Low-dose ICS	High-dose ICS	
	Frequency (%)	Frequency (%)	p-value
Total number included for analysis	22 (9 BDP / 7 BUD / 6 FLU)	33 (16 BUD / 17 FLU)	
Mean age (range)	58.9 (14.4) (21–72)	54.1 (14.3) (25–80)	0.22
Sex: male	12 (54.6)	14 (41.2)	0.33
Ethnic group:			
Black/Black British	0	2 (6.1)	
White/White British	22 (100)	31 (93.9)	0.70
Smoking history:			
Ex-smokers	7 (31.8)	11 (32.4)	
Non smokers	15 (68.2)	22 (67.7)	0.97
Current eczema	4 (18.2)	3 (9.1)	0.42
Current hay fever	10 (45.5)	13 (39.4)	0.59
≥1 severe asthma exacerbation in last year	0 (0)	4 (12)	0.28
**Inhaled treatment**			
ICS only	13 (59.1)	3 (9.1)	
ICS + LABA	9 (40.9)	30 (90.9)	
	**Mean (SD)**	**Mean (SD)**	**p-value**
ICS dose (BDP equivalent μg)*	400 (200)*	1000 (200)*	
ACQ score	0.9 (0.6)	1.2 (0.8)	0.12
LCQ score*	19.0 (2.0)*	17.8 (3.5)*	0.08
FEV_1_% predicted	93.5 (28.0)	93.1 (22.5)	0.95
FEV_1_/FVC ratio %	68.8 (10.1)	71.2 (11.4)	0.43
Blood eosinophil count (x10^9^ /L)* (peak in last 12 months)	0.2 (0.2)	0.3 (0.325)	0.41
FE_NO_ concentration (ppb)†	17.2 (12.8–23.1) †	13.0 (9.0–18.6) †	0.27‡
Sputum bacterial load (cfu/mL)*	1.35x10^7^ (9.89x10^7^)	8.86x10^6^ (2.81x10^7^)	0.27

*Figures shown are median and IQR

†Figures shown are geometric mean and 95% CI

‡ T-test comparing log FE_NO_

ICS: inhaled corticosteroids, LABA: long-acting β_2_-agonist, BDP: beclomethasone dipropionate, BUD: budesonide, FLU: fluticasone propionate, ACQ: Asthma Control Questionnaire, LCQ: Leicester Cough Questionnaire, FEV_1_: forced expiratory volume in 1 second, FVC: forced vital capacity, FE_NO_: exhaled nitric oxide.

**Table 2:** Frequency distribution of patient groups attending visit 1 and visits 2 and 3 based on treatment group.

Treatment group	ICS	Visit 1 (n =)	Visit 2 (24 h) and 3 (2 weeks) (n =)
Low-dose ICS (n = 22)	BDP (≤400 μg/day)	10	6
	BUD (≤400 μg/day)	7	2
	FP (≤200 μg/day)	5	0
High-dose ICS (n = 33)	BUD (≥800 μg/day)	16	4
	FP (≥500 μg/day)	17	7
Total		55	19

BDP: Beclomethasone dipropionate; BUD: Budesonide; FP: Fluticasone propionate; ICS: inhaled corticosteroid.

#### 16S rRNA gene sequencing

**Fig 1:**
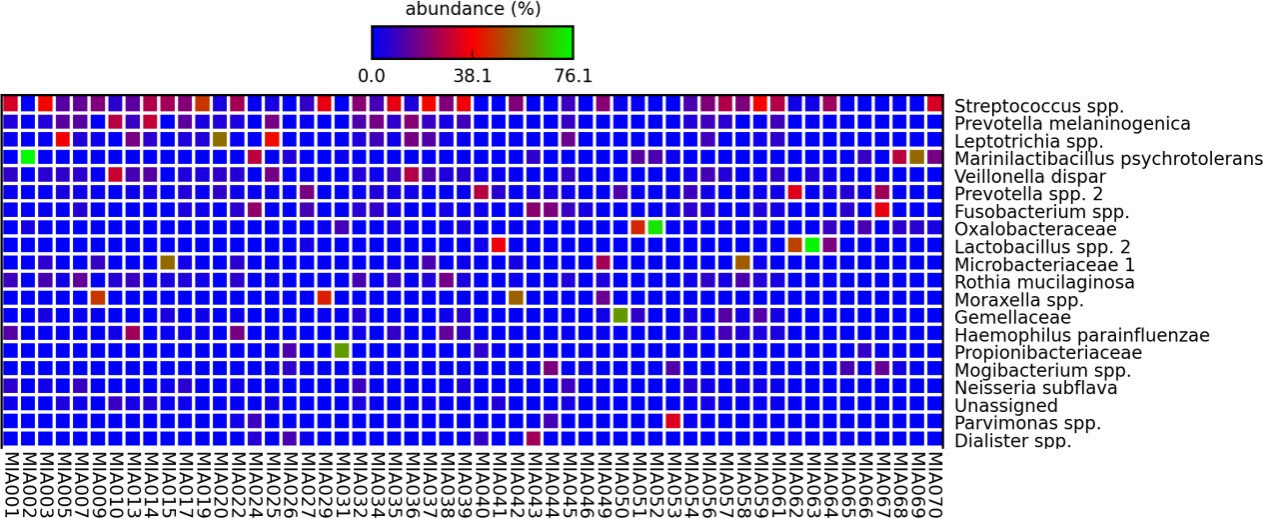
Top 20 OTUs identified from 54 patients, with the highest mean abundance (%) being the topmost. The colours indicate the abundance of OTUs in each patient, with blue regarded as low abundance, red as moderate abundance and green as high abundance.

A total of 5,615,037 sequencing reads were generated from 54 of 55 asthma patients. The paired end reads were rarefied to 9311 reads resulting in one patient sample being excluded due to low reads (4693 reads). Sequences were clustered into 167 OTUs. The OTUs *Streptococcus* spp. and *Prevotella* spp. were detected in all patients with high mean abundance (%) (Table 3 and Fig 1). Despite the large number of OTUs identified from this cohort, only 11 OTUs were found in ≥90% of the patients. These included *H*. *parainfluenzae*, which was found in 90.7% patients. Based on a previous classification [25], OTUs were divided into aerobes/anaerobes (Table 3). Among the most prevalent OTUs, 27% were identified as aerobes, with *Streptococcus* spp. the most common. Among other OTUs found from this cohort were the genera *Moraxella*, *Pseudomonas* and *Staphylococcus*. Though *Moraxella* spp. accounted for only 33% of the cohort, 4 patients (aged 68–71 years) had a very high relative abundance of the genus (16.17–52.59%). *S*. *aureus* was identified in 61% of patients; only one patient had this taxon as a high relative abundance (17.65%) with others below 4%. *Pseudomonas* spp. was found in 81% of the cohort, with all relative abundances below 4%.

**Table 3:** The most prevalent OTUs across samples (90–100% patients) in descending order of mean abundance (%).

OTU	Mean abundance (%)	Relative abundance ranges (%)	Oxygen requirements
*Streptococcus* spp.	14.13	0.01–45.85	Aerobe
*Veillonella dispar*	4.60	0.003–30.33	Anaerobe
*Prevotella* spp.	4.02	0.003–33.82	Anaerobe
*Fusobacterium* spp.	3.73	0.009–34.69	Anaerobe
Oxalobacteriaceae	3.58	0.001–72.44	Both
*Haemophilus parainfluenzae*	2.58	0.001–24.49	Aerobe
*Mogibacterium* spp.	1.43	0.001–18.85	Aerobe
Lachnospiraceae	1.03	0.001–10.16	Anaerobe
*Atopobium* spp.	0.77	0.002–12.21	Anaerobe
*Selenomonas* spp.	0.63	0.001–9.26	Anaerobe
*Prevotella pallens*	0.45	0.001–4.87	Anaerobe

#### Correlation of lung microbiota with ICS dose

**Fig 2:**
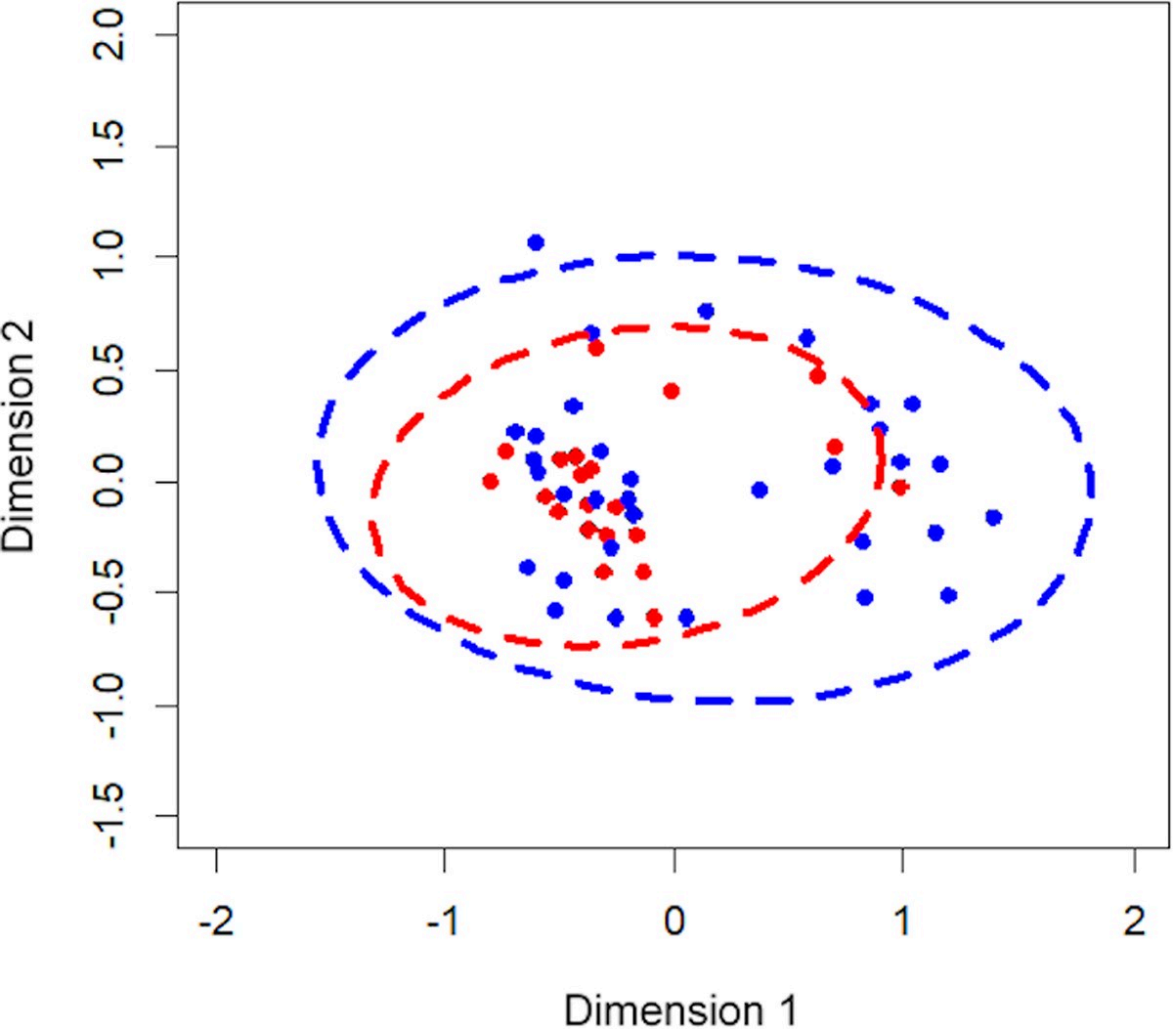
The Non-metric Multidimensional Scaling (NMDS) plot represented the bacterial composition of each patient, grouped by ICS dose; low-dose ICS (red) and high-dose ICS (blue). The shorter the distance between the dots, the more similar the bacterial compositions were between the patients.

ICS dose (high or low) did not affect species richness (p = 0.23), diversity (Simpson’s Diversity Index, p = 0.134), or community structure (Shannon’s index, p = 0.256). The microbiota composition in patients from the low- and high-dose ICS groups is shown in Fig 2. No significant differences were observed in the microbiota composition between the groups (Bray-Curtis; p = 0.676).

Load changes of individual OTUs between ICS dose groups are presented in Table 4. In total, 32 OTUs were significantly more prevalent in low-dose ICS patients. Among these were 5 of the most prevalent OTUs from the cohort as a whole (*Streptococcus* spp., *V*. *dispar*, *Atopobium* spp., *Selenomonas* spp. and *P*. *pallens*; Table 3). *Streptococcus* spp. was the most abundant OTU detected in the low-dose ICS group (2.6 x 10^7^ CFU/ml), at significantly higher levels than were found in the high-dose ICS group (1.72 x 10^6^ CFU/ml, p = 0.002). Three other members of the *Prevotella* genus (besides *P*. *pallens*) were also found to be more abundant in the low-dose ICS group, namely *P*. *tannerae*, *P*. *melaninogenica* and *P*. *nigrescens* (Table 4). Most of the more abundant organisms in low-dose ICS patients were anaerobes. Only 7 OTUs were significantly higher in high-dose ICS patients but with lower mean abundances than those more frequent in the low-dose group (*Dysgonomonas* spp., *Parvimonas* spp.).

**Table 4:** Significant OTU changes between ICS dose groups.

OTU	Low-dose ICS	High-dose ICS	p-value
	Mean (CFU/ml)*	Mean (CFU/ml)*	
*Acidocella* spp.	3.77E+04 **↑**	3.29E+03	0.011
*Actinomyces* spp.	2.40E+06 **↑**	1.78E+05	0.02
*Aggregatibacter segnis*	4.06E+05 **↑**	2.35E+04	0.043
*Atopobium* spp.	6.14E+05 **↑**	8.16E+04	0.006
Bacillaceae 1	2.27E+04	2.62E+05 **↑**	0.047
Bacillaceae 2	1.73E+04	1.82E+05 **↑**	0.031
*Campylobacter* spp.	7.13E+05 **↑**	2.26E+05	0.048
*Capnocytophaga* spp.	4.09E+05 **↑**	1.28E+04	0.005
*Cardiobacterium* spp.	3.00E+04 **↑**	6.50E+02	0.019
*Catonella* spp.	1.46E+05 **↑**	2.56E+04	0.048
Cerasicoccaceae	0.00E+00	4.81E+04 **↑**	0.045
*Corynebacterium durum*	2.51E+04 **↑**	6.90E+03	0.031
*Dialister* spp.	7.35E+05 **↑**	1.39E+05	0.016
*Dysgonomonas* spp.	5.72E+02	5.32E+04 **↑**	0.008
Enterobacteriaceae	1.89E+01	6.52E+03 **↑**	0.032
*Granulicatella* spp.	1.23E+06 **↑**	6.41E+04	0.015
*Lautropia* spp.	7.41E+05 **↑**	2.09E+04	0.039
*Leptotrichia* spp.	1.08E+07 **↑**	2.19E+06	0.003
*Megasphaera* spp.	3.83E+05 **↑**	5.73E+04	0.009
Microbacteriaceae	8.97E+05	9.13E+05 **↑**	0.012
*Moryella* spp.	8.02E+05 **↑**	6.00E+04	0.001
Neisseriaceae	2.42E+05 **↑**	5.25E+03	0.007
*Oribacterium* spp.	7.87E+05 **↑**	1.60E+05	0.006
*Parvimonas* spp.	5.02E+05	7.60E+06 **↑**	0.005
*Prevotella melaninogenica*	1.07E+07 **↑**	1.57E+06	0.016
*Prevotella nigrescens*	1.53E+05 **↑**	4.77E+03	0.008
*Prevotella pallens*	7.37E+05 **↑**	1.77E+05	0.029
*Prevotella tannerae*	5.67E+05 **↑**	3.72E+04	0.031
*Rothia aeria*	6.17E+05 **↑**	1.99E+04	0.005
*Rothia dentocariosa*	8.44E+05 **↑**	6.31E+04	0.01
*Rothia mucilaginosa*	1.40E+07 **↑**	6.09E+05	0.021
*Selenomonas noxia*	2.91E+04 **↑**	5.21E+02	0.028
*Selenomonas* spp.	6.82E+05 **↑**	3.10E+05	0.006
*Streptococcus anginosus*	9.52E+04 **↑**	1.17E+04	0.002
*Streptococcus* spp.	2.60E+07 **↑**	1.72E+06	0.002
TM_7.Rs_045	1.38E+05 **↑**	1.34E+03	0.035
TM7	5.68E+05 **↑**	1.44E+04	0.002
*Veillonella dispar*	8.80E+06 **↑**	1.09E+06	0.013
>Weeksellaceae	>5.80E+04 **↑**	>4.62E+03	0.002

Group with highest comparative abundance of each OTU signified by ↑.

* normalised OTU relative abundance (%) were multiplied by their corresponding qPCR-derived total bacterial load obtained from the qPCR data to give a CFU/ml value.

#### Correlating lung microbiome with type of high-dose ICS

**Fig 3:**
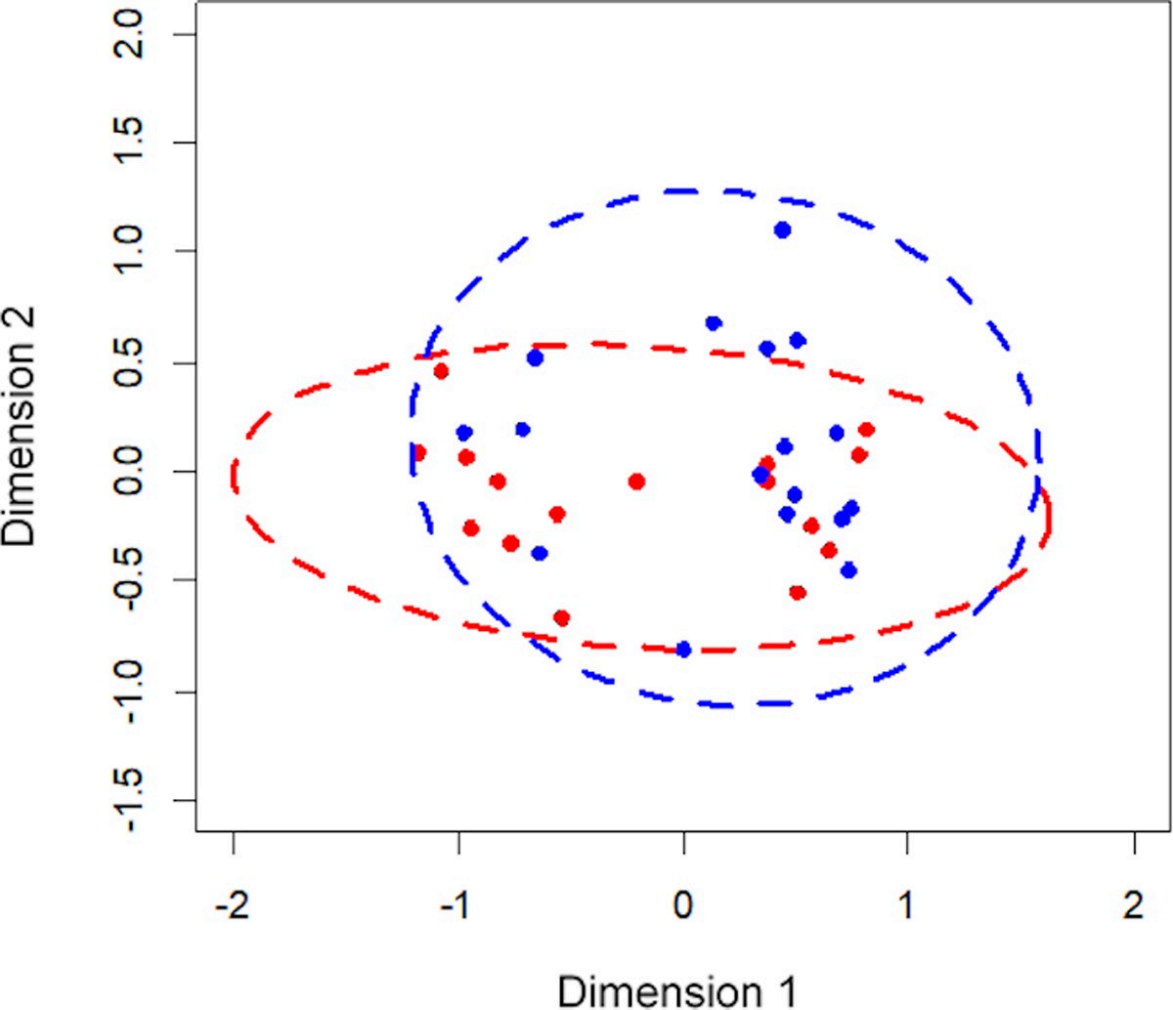
The Non-metric Multidimensional Scaling (NMDS) plot based on bacterial composition of patients on high-dose ICS taking budesonide (red) and fluticasone propionate (blue). The shorter the distance between the dots, the more similar the bacterial compositions were between the patients.

Species richness was not significantly different between high-dose FP and BUD treated subjects (p = 0.9). Similarly, non-significant values were evident for Simpson’s Diversity Index (p = 0.787) and community structure (p = 0.887). The microbiota composition in patients from different types of high-dose ICS groups is shown in Fig 3. The Bray-Curtis distances between groups showed no significant differences in bacterial composition (p = 0.345).

#### *Haemophilus* and high-dose ICS

A total of 9 OTUs had significantly higher mean abundance in the high-dose FP group (Table 5). Among these, *H*. *parainfluenzae* had a significantly higher abundance in individuals taking high-dose FP (mean abundance: 9.64 x 10^5^ CFU/ml) than those taking high-dose BUD (1.45 x 10^5^ CFU/ml, p = 0.047). This led to the qPCR investigation to determine *H*. *influenzae* and *S*. *pneumoniae* loads in high-dose FP and BUD groups (described in S1 File and S1 Fig), which determined *H*. *influenzae* loads were significantly higher than *S*. *pneumoniae* in both high-dose FP and BUD groups but found no significant differences in *H*. *influenzae* or *S*. *pneumoniae* loads between these groups.

**Table 5:** OTU changes between type of ICS in the high-dose ICS group.

OTU	FP (n = 17)	BUD (n = 16)	p-value
	Mean (CFU/ml)	Mean (CFU/ml)	
*Anaerobacillus* spp.	1.00E+05 **↑**	8.26E+04	0.026
*Capnocytophaga ochracea*	1.07E+04 **↑**	1.22E+03	0.038
*Dysgonomonas* spp.	5.33E+04 **↑**	5.31E+04	0.035
*Exiguobacterium* spp.	3.12E+02	4.43E+03	0.026
*Fluviicola* spp.	1.38E+01	1.26E+03	0.043
*Haemophilus parainfluenzae*	9.64E+05 **↑**	1.45E+05	0.047
*Lactobacillus reuteri*	2.85E+04 **↑**	0.00E+00	0.027
*Paracoccus* spp.	9.00E+04	1.95E+05	0.016
*Peptococcus* spp.	1.86E+04 **↑**	6.93E+01	0.014
*Porphyromonas endodontalis*	6.40E+04 **↑**	6.01E+03	0.009
*Rhodobaca* spp.	1.80E+04	1.90E+04	0.049
Veillonellaceae	1.18E+04 **↑**	5.81E+00	0.048
Xanthomonadaceae	7.18E+04 **↑**	5.31E+03	0.019

BUD: Budesonide; FP: Fluticasone propionate, ICS: inhaled corticosteroid.

**↑** higher in FP compared to BUD.

#### Changes in microbiota over time

**Fig 4:**
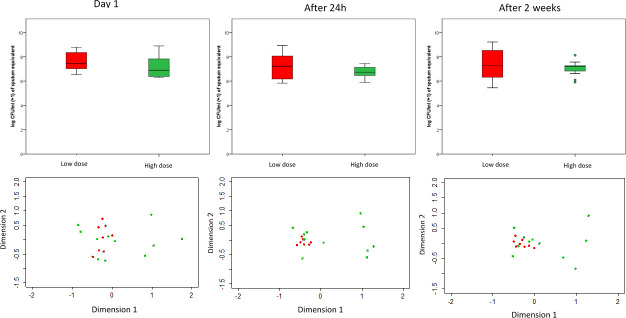
The NMDS (non-metric multidimensional scaling) of the microbiota composition of asthma patients (top) and total bacterial load (log CFU/ml +1) (bottom) grouped into ICS dose group of low-dose ICS (red) and high-dose ICS (green) at (a) Day 1, (b) Day 2 (after 24 h) and (c) Day 14 (after 2 weeks).

Both analyses on microbiota composition and qPCR total bacteria data between different time points (Day 1, 2 and 14) of 19 patients demonstrated no significant changes in diversity measures and bacterial load (Fig 4). The overall composition was measured by species richness, diversity (Simpson’s) and community structure (Shannon’s) with no significant changes between different time points.

## Discussion

This study presents a culture-independent analysis of bacteria present in respiratory samples of adults with clinically stable asthma and provides several important and novel findings to the field of asthma microbiome studies. All individuals had been taking the same type and dose of ICS for at least a year allowing the impact of long-term usage to be evaluated. A number of patients were sampled more than once allowing insights into short-term asthma microbiota stability.

From the 167 OTUs identified, a restricted number (eleven) were found in over 90% of individuals studied. This is in keeping with findings for the cystic fibrosis airways microbiome in which core and transient populations have been established [25]. In this study, the taxa identified often matched those previously found in asthma and in cystic fibrosis and bronchiectasis such as *Streptococcus* spp., *Haemophilus* spp. and anaerobes (*Veillonella* spp., *Prevotella* spp.). These have been detected in poorly-controlled asthma in adults [16, 26], treatment-resistant severe asthma in adults [14] and mild [27] and severe asthma patients [10]. Of these genera, *Haemophilus* and *Streptococcus* include species considered in other contexts to be pathogens. The consequence of identifying putative pathogens in the asthmatic airways raises important clinical questions as to their potential contribution to the pathophysiology of asthma and also whether or not to treat potential pathogens when detected by such technologies. In relation to this, it is important to consider the bacterial load carried in the asthmatic airways. The average sputum bacterial load of this cohort (~1x10^7^ CFU/mL) is between that observed in healthy controls (<1x10^6^ CFU/mL) [28] and in cystic fibrosis (~1x10^8^ CFU/mL) [29]. As such, the potential for pathophysiological airway damage is a distinct possibility in asthmatic individuals.

The relationship between ICS dose and lung microbiota composition was explored. The clinical characteristics of the low- and high-dose ICS groups were similar with no significant differences in FEV_1_, FE_NO_ or peak blood eosinophil count/number of severe asthma exacerbations in the last year. In general, ICS dose did not affect ecological community characteristics. In contrast, previously, microbiota from severe asthmatics (taking a similar dose of ICS to our high-dose group) was enriched in 53 genera compared to mild/moderate subjects, the majority being *Actinobacteria*, with the remaining 5 classifying to *Gammaproteobacteria* [30]. The differences between previous results and our findings may be due in part by fact that Huang *et al*. sampled the microbiota via bronchial brushings. It is also possible that the severe asthma group from this study had sub-optimal asthma control whilst our high-dose ICS group were generally well-controlled.

A number of genera had significantly higher relative abundances in low-dose ICS patient samples with a smaller number of OTUs detected that were more abundant in the high-dose group. The most prevalent OTU identified in low-dose samples was *Streptococcus* spp. This genus contains species that differ in their potential to cause pathophysiological damage and without extensive subsequent analysis it is not possible to assess whether the Streptococcal species being detected are pathogens or not. Cox *et al*. [31] previously reported that 16S rRNA gene sequencing was unable to discriminate between *S*. *pneumoniae* and *Streptococcus mitis*, the latter being a normal commensal of the oropharynx. A smaller set of genera were more abundant in the high-dose ICS group. Of these, the most prevalent was *Parvimonas* spp. a genus that contains species regarded as human pathogens [32]. The significance of the differing abundances of these genera between high- and low-dose ICS groups is however unclear. These differences may be part of a process which is either a cause or consequence of disease progression. It is also possible that ICS act as “selecting agents” for certain species. In many studies high doses of specific compounds are associated with selection of a few distinct taxa [33].

No significant differences in overall microbiota composition or bacterial load were identified between patients taking high-dose BUD and high-dose FP. However a higher abundance of *H*. *parainfluenzae* was identified in the high-dose FP group than in those using high-dose BUD. A selective effect of long-term FP usage on the abundance of this potential pathogen is therefore a possibility. Goleva *et al*. [34] found an increased abundance of *H*. *parainfluenzae* in asthmatics “resistant” to a treatment trial of oral prednisolone in comparison to those who were steroid “sensitive” and an inhibitory effect of *H*. *parainfluenzae* on asthmatic airway macrophages *in vitro* was also observed. The differences in abundance of other organisms between high-dose BUD and FP are of uncertain significance and include some organisms usually associated with oral (*Veillonellaceae*, *Porphyromonas endodontalis*) and GI (*Lactobacillus reuteri*, *Peptococcus* spp.) microbiota.

Evidence is accumulating that ICS treatment is associated with an increased risk of pneumonia in COPD [6, 35] and respiratory infection in asthma [4, 5] and that this increased risk is greater with FP [36] than BUD. Our findings, combined with the observation that short-term treatment with FP can cause distinct changes in the microbiota [37], provide a possible mechanism but a prospective study of the microbiota of patients starting different ICS is really required to strengthen these observations.

This study also examined the short-term stability of the asthma microbiota. No statistically significant differences were seen in community composition between the three time points over two weeks, suggesting the asthma microbiome is typically stable over short periods of time in clinical stability.

This study has limitations. Microbial contamination, possible from the oral cavity, has been covered extensively elsewhere [38]. No evidence for gross contamination was observed here with the commonality of species detected across over 90% of individuals with asthma supportive of non-contaminated sample collection. A further limitation could be considered the lack of subject phenotyping. Distinct asthma phenotypes may have distinct airways microbiota [39]. This will need to be considered when designing future studies. Also, the mean age of our patients was higher than previous studies [10, 27, 30]. Whilst the effect of age on the microbiota has not previously been investigated, data from CF patients suggest a decrease in microbiota diversity with age [40]. Finally, a “molecular” limitation was that the differences in taxa were described primarily at genus level making the biological effects less clear.

## Conclusions

This study did not demonstrate a significant difference in microbiota composition between asthma patients taking low- and high-dose ICS. However, an association was identified between high-dose FP and increased abundance of the pathogen *H*. *parainfluenzae*. The clinical implications for patients are not known but this does provide a possible explanation for the increased risk of pulmonary infection seen in asthma and COPD, particularly with FP.

## Supporting information

S1 File (DOCX)

S1 Data (XLSX)

S2 Data (XLSX)

S1 Fig Total bacteria, *H*. *influenzae* and *S*. *pneumoniae* levels (log CFU/ml +1) in subjects receiving high-dose inhaled corticosteroids: Budesonide (BUD) and fluticasone propionate (FP). (TIF)

## Abbreviation list

16S rRNA: 16S ribosomal ribonucleic acid
ANOSIM: Analysis of Similarity
ANOVA: Analysis of Variance
BDP: Beclomethasone dipropionate
BUD: Budesonide
CFU: Colony Forming Units
COPD: Chronic Obstructive Pulmonary Disease
DNA: Deoxyribonucleic acid
FE_NO_: Fractional Exhaled Nitric Oxide
FEV_1_: Forced Expiratory Volume in 1 second
FP: Fluticasone propionate
ICS: Inhaled corticosteroids
LABAs: Long-Acting Beta Agonists
LAMAs: Long-Acting Muscarinic Antagonists
NMDS: Non-metric multidimensional scaling
OTU: Operational Taxonomic Units
qPCR: Quantitative Polymerase Chain Reaction
