# Supplementary material for: The airways microbiome of individuals with asthma treated with high and low doses of inhaled corticosteroids
Source: PLoS One. 2020 Dec 30;15(12):e0244681. doi: 10.1371/journal.pone.0244681 (PMC7773270; doi:10.1371/journal.pone.0244681)
Supplement: S1 File — (DOCX) [file pone.0244681.s001.docx]

## A1 Diagnosis of asthma

All participants in the study had a physician diagnosis of asthma. Diagnoses of asthma were confirmed with positive bronchodilator reversibility of ≥12% and ≥200 mL in FEV_1_ or provocative concentration of methacholine (PC_20_) of ≤8 mg/mL if available).

## A2 Clinical measurements

Spirometry was performed using a Vitalograph™ dry wedge bellows spirometer (Vitalograph™ model 2150, Buckinghamshire, England) and the FEV_1_ and FVC were calculated. FE_NO_ concentration was measured using an offline electrochemical analyser (NOBreath®, Bedfont Scientific Ltd, Harrietsham, UK) at a flow of 50mL/s.

Prior to sputum induction in order to reduce oral microbiota contamination, separation of saliva from sputum was achieved by asking the patients to mouth rinse with 10 ml 0.9% sterile saline prior to nebulisation with hypertonic saline. The induced sputum samples were collected into sterile specimen containers. Prior to storage, 50 mg of each sample was homogenised with dithiothreitol (DTT) (4 µl of 0.1% DTT per mg sputum) and rapidly frozen at -80 °C.

## A3 DNA extraction method

Sputum samples (100 µL) were incubated for 30 min at 37 ^o^C with lysozyme (200 µL; 45 mg/mL, Sigma-Aldrich Co. Ltd., Dorset, UK), suspended in Gram-Positive Lysis Solution and processed following the recommendations for Gram positive bacterial DNA extraction (GenElute™ Bacterial Genomic DNA Kit). Cell disruption was then achieved by insertion of tungsten carbide and glass beads, followed by agitation in a Fastprep-24 Instrument (MP Biomedicals Europe, Illkirch, France) at 6.5 m/s for 60 sec. After processing, DNA was resuspended in 50 µL of Elution Solution (GenElute™ Bacterial Genomic DNA Kit). Sterile PBS (100 µL) was used as negative control with no genomic DNA detected when assessed by spectrophotometry (Picodrop Microlitre Spectrophotometer, GRI, Braintree, UK).

## A4 16S rRNA gene sequencing

The size of the library was measured using the Agilent High Sensitivity DNA kit (Agilent, Germany) and quantified using ABI Viaa7 and KAPA Library Quantification Kit Illumina® platforms (KAPABiosystems). The sequencing was then performed on the MiSeq platform (Illumina, USA) using the MiSeq reagent kit V2 (500 cycles) according to manufacturer’s instructions. The raw sequence data obtained from the Illumina MiSeq sequencer were filtered to remove any chimeric sequences from the input sequences, which could present at a low level due to premature amplicon termination during the library preparation step. The paired end reads were rarefied to 9311 reads followed by analysis based on the Operational Taxonomic Unit (OTU) approach. This was determined using the QIIME version 1.9.1 pipeline to cluster the 16S rRNA gene sequences based on their similarity. Within these data, a total of 5615037 sequencing reads were clustered into a final 167 OTUs, where OTUs less than 0.01% relative abundance across all samples sets were discarded. One sample was removed from the whole OTU analysis due to low sequence reads (4693 reads).

## A5 qPCR

**Total Bacterial Load (TBL) qPCR**

Total bacterial load was estimated by using the SYBR Green dye, using the primers EubF 5’-TCCTACGGGAGGCAGCAGT-3’ and EubR 5’-GGACTACCAGGGTATCTA ATCCTGTT-3 (Sigma-Aldrich Co. Ltd.) which amplified a 466-bp region between positions 331 to 797 of the *Escherichia coli* 16S rRNA gene ^38^. The assay was performed as described in ^39^. All PCR reactions were carried out in a total volume of 20 μl containing primers at a concentration of 500 nM each, 1 μl of template and Rotor-Gene SYBR Green PCR Master Mix (Qiagen) at 1x final concentration. Quantitative PCR assay was performed using Rotor-Gene Q real-time thermocycler (Qiagen) with a temperature profile of 95 °C for 5 min, followed by 50 cycles at 95 °C for 15 s and 58 °C for 50 s. Gain optimisation was set manually at 5.33 on the green channel (Cycling A. Green). Melt-curve analysis was then conducted between 58°C to 99°C with 1 °C steps, to detect non-specific amplifications.

***H. influenzae* (HI) qPCR**

*H. influenzae* densities were estimated by TaqMan assay, using the primers HelSF 5’-CCGGGTGCGGTAGAATTTAATAA-3’, HelSR 5’-CTGATTTTTCAGTGCTGTCTTTGC-3’ (Eurofins Genomics) and probe HelSPr 5’-FAM-ACAGCCACAACGGTA AAGTGTTCTACG-TAMRA-3’ (Eurofins Genomics) which amplify a 90-bp region between positions 518 to 608 of the *H. influenzae* hel gene ^20^. All PCR reactions were carried out in a total volume of 20 μl containing primers and probe at a concentration of 500:500:250 nM (HelSF: HelSR; HelSPr), 1 μl of template and LightCycler 480 Probes Master (Roche Diagnostics GmbH, Mannheim, Germany) at 1x final concentration. Quantitative PCR assays were carried out using the Rotor-Gene Q (Qiagen) with a temperature profile of 95 °C for 5 min, followed by 45 cycles at 95 °C for 15 s and 60 °C for 60 s. Gain setting on the green channel was optimised manually to 4.00 for each run. The lowest detection limit of this assay was 100 CFU/ml.

***S. pneumoniae* (SPN) qPCR**

The assay was performed using a TaqMan based probe, lytA-CDCPr 5′-FAM-TGCCGAAAACGCTTGATACAGGGAG- BHQ1-3′ (Eurofins Genomics) and primers lytA-CDCF 5′-ACGCAATCTAGCAGATGAAGCA-3′, lytA-CDCR 5′-TCGTGCGTTTTAATTCCAGCT-3′ (Eurofins Genomics). A 53-bp region was amplified between positions 1840961 to 1841014 of the *S. pneumoniae* lytA gene ^21^. Primers and probe concentrations were optimised to produce an assay with a final 25 µL reaction volume as follows: Primers and probe at a concentration of 500:500:250 nM (*lytA*-CDCF: *lytA*-CDCR: *lytA*-CDCPr), 1 µL of template and LightCycler 480 Probes Master (Roche Diagnostics GmbH) at 1x final concentration. Quantification of DNA copies was performed using the Rotor-Gene Q (Qiagen) with a temperature profile of 95 °C for 5 min, followed by 45 cycles at 95 °C for 15 s and 60 °C for 60 s. Gain setting on the green channel was set manually to 4.00 for each run. The lowest detection limit of this assay was 100 CFU/ml.

**A6 *H. influenzae* (HI) and *S. pneumoniae* (SPN) qPCR results**

*H. influenzae* was detected in 6 of 17 individuals taking high-dose FP, ranging from 1.62 x 10^2^ to 3.94 x 10^7^ CFU/ml, including 4 patients less than 100 CFU/ml. *H. influenzae* was also detected in half of the high-dose BUD treated group, in which 7 patients had levels ranging between 1.1 x 10^2^ to 2.52 x 10^5^ CFU/ml and 1 patient with less than 100 CFU/ml.

*H. influenzae* loads were significantly higher than *S. pneumoniae* in both high-dose ICS groups (BUD p=0.012, FLU p=0.017) (Figure 4). *S. pneumoniae* was markedly less abundant in both groups, where only 3/16 and 4/17 patients taking BUD and FP respectively, were positive for the species but at levels below 1.00 x 10^2^ CFU/ml.
