# Supplementary figures and images for: The airways microbiome of individuals with asthma treated with high and low doses of inhaled corticosteroids
Source: PLoS One. 2020 Dec 30;15(12):e0244681. doi: 10.1371/journal.pone.0244681 (PMC7773270; doi:10.1371/journal.pone.0244681)

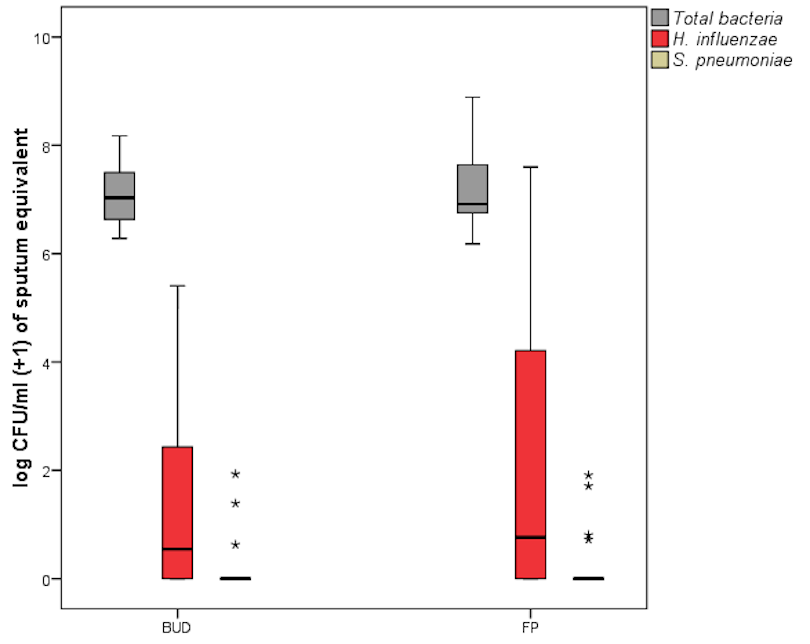

Supplement: S1 Fig — (TIF) [file pone.0244681.s004.tif]
